# Asymptomatic malaria parasitaemia and virological non-suppression among children living with HIV in Accra, Ghana: a cross-sectional study

**DOI:** 10.21203/rs.3.rs-3823525/v1

**Published:** 2024-01-09

**Authors:** Adwoa K. A. Afrane, Yakubu Alhassan, Linda Eva Amoah, Mame Yaa Nyarko, Adolphina Addo-Lartey, Elijah Paintsil, Kwasi Torpey

**Affiliations:** Department of Child Health, University of Ghana Medical School; Department of Biostatistics, School of Public Health, University of Ghana, Legon; Department of Immunology, Noguchi Memorial Institute for Medical Research, University of Ghana; Princess Marie Louise Children’s Hospital; Department of Epidemiology and Disease Control, School of Public Health, College of Health Sciences, University of Ghana; Boston University Chobanian and Avedisian School of Medicine, Boston; Department of Population, Family and Reproductive Health, School of Public Health, College of Health Sciences, University of Ghana Legon

**Keywords:** Antiretroviral therapy, asymptomatic malaria, parasitaemia, Paediatric HIV, viral load, virological non-suppression

## Abstract

**Background:**

Human Immunodeficiency Virus (HIV) and malaria are two major diseases in sub-Saharan Africa, with coinfections having an impact on the outcomes of both. We assessed the association between asymptomatic malaria parasitaemia and virological non-suppression among children living with HIV attending a clinic at the Korle Bu Teaching Hospital (KBTH) and the Princess Marie Louis Hospital (PML) in the city of Accra, Ghana.

**Methods:**

This was a cross-sectional study of asymptomatic malaria in children receiving care at paediatric HIV clinics at KBTH and PML conducted from September to November 2022. Patients who had been on ART for at least 6 months were eligible to participate. Structured questionnaires were used to collect socio-demographic, malaria prevention behaviors, and ART-related data using in-person interviews. Microscopy and PCR were used to screen for malaria and GeneXpert to determine viral load. To examine the determinants of malaria PCR positivity and virological non-suppression, Chi-square tests and logistic regression were utilized.

**Results:**

The participants’ median age was 9 years with a range of 6 to 12 years. Males made up 57% of the population. We detected 3.6% (10 of 277) and 7.6% (21 of 277) cases of malaria using microscopy and PCR, respectively. Virological non-suppression (VL > 1000 copies/ml) was seen in 82 (29.6%) of the 277 participants. Among the suppressed individuals, 62 (22.4%) exhibited low-level viraemia (VL level 40–1000 copies/ml) and 133 (48%) had non-detectable viral load levels. There were no factors associated with malaria PCR positivity carriage. Poor adherence to antiretroviral therapy was associated with a fivefold increase in the risk of viral load non-suppression (AOR = 4.89 [CI = 2.00–11.98], p = 0.001).

**Conclusion:**

The study showed that the proportion of children living with HIV with asymptomatic malaria parasitaemia was low, with about one third of the study population having virological non suppression. The interaction between malaria parasitemia and viral replication may not be the main culprit for virological non suppression.

## BACKGROUND

Human immunodeficiency virus (HIV) is an infectious disease pathogen that causes cellular immune system impairment [1]. Sub-Saharan Africa (SSA) accounts for more than half of all HIV infections and AIDS-related deaths worldwide (WHO, 2021). In addition, approximately 213 million malaria infections and 380,000 deaths are reported annually in Africa according to the World Health Organization (WHO). Given that HIV infection weakens the immune system, infected people, particularly children, are more vulnerable to other infections such as malaria [2]. Simultaneous infection by HIV and malaria may be as high as 30% in children living with HIV (CLHIV) in SSA [1], [2].

In children, more frequent episodes of clinical malaria have been observed in CLHIV compared to HIV-uninfected children, and this increases with the severity of immunosuppression [3]. CLHIV are also at a higher risk of having severe malaria and an impaired response to antimalarial treatment as compared to HIV-uninfected children. This however depends on factors such as immunosuppression level, and previous immunity to malaria [3]. The presence of malaria infection in CLHIV provides an ideal microenvironment for rapid HIV-1 replication due to up-regulation of pro-inflammatory cytokines [4]. There is also strong CD4 + cell activation and spread of the virus among CD4 + cells due to the malaria parasitemia. In effect the increased concentrations of HIV-1 RNA in the blood result in increased disease progression and is correlated with a higher risk of transmission of the virus by blood, vertical and sexual means [5].

The use of preventive cotrimoxazole and insecticide-treated bed nets lowers the risk of malaria in CLHIV. This however, does not offer complete protection against malaria and thus, the burden of malaria is still high in areas of high-intensity transmission [6]. Asymptomatic malaria is defined as “the presence of parasites in the blood of an individual in the absence of malaria-related symptoms such as fever, i.e., temperature greater than 37.5°C” [7]. Studies have shown that in areas of intense malaria transmission, such as Ghana, asymptomatic children are major carriers of *Plasmodium falciparum* and, therefore, contribute to the parasite reservoir [8][9]. To reduce the overall and area-specific malaria transmission in countries with high malaria transmission, there is a need for data on the epidemiology of asymptomatic malaria. This information would help programs control the effect of local malaria transmission and associated factors.

The ultimate goal of combination ART (cART) for CLHIV is to achieve long-term virological suppression [10]. In low- and middle-income countries, a viral load (VL) < 1000 copies/ml defines treatment success (suppression), which also indicates treatment adherence and reduced risk of HIV transmission [11]. There are however various factors that influence virological suppression which include socio-demographic factors, HIV comorbidities, HIV clinical severity, and ART regimen. There is a need to identify these factors in order to help target interventions in the affected population that would ultimately lead to improvement in clinical care.

Surveys to characterize baseline malaria transmission epidemiology are recommended by the WHO. These surveys identify those carrying malaria parasites and populations at risk such as CLHIV to help develop targeted control measures for a better impact [12]. In developing countries such as Ghana, patient care and public health policy are greatly affected by the clinical, diagnostic, and therapeutic interactions between HIV and malaria. This study therefore aimed to determine the proportion of CLHIV with asymptomatic malaria parasites and factors associated with virological non-suppression at Korle Bu Teaching Hospital (KBTH) and Princess Mare Louis (PML) Hospital.

## METHODS

### Study Design and Setting

A cross-sectional study was conducted at Korle Bu Teaching Hospital (KBTH) and Princess Marie Louis (PML) Hospital, in the city of Accra, Greater Accra region of Southern Ghana. Recruitment took place from September to November 2022. The Paediatric HIV clinic at KBTH has been providing comprehensive HIV/AIDS care and management services since 2004. An average of 40 patients are seen per clinic day which is run once a week. Patients are referred from primary and secondary health facilities as well as from other departments within the hospital. Children are seen from 6 weeks of age till approximately fifteen years of age when they would have had full disclosure and are ready to be transferred to the adolescent HIV clinic at KBTH. The HIV clinic at PML sees approximately 10–20 patients every week and the clinic is run twice a week. Referrals to the PML Paediatric HIV clinic are mainly from the southern part of Ghana. The PML is a children’s hospital that mainly sees paediatric patients up to 18 years of age.

As per the National AIDS Control Program (NACP) guidelines, VL is obtained at least once a year per patient and the cost is borne by NACP. National treatment guidelines updated with WHO recommendations are used by both HIV clinics. Antiretroviral drugs that are provided at the clinics are abacavir (ABC), nevirapine (NVP), lamivudine (3TC), tenofovir (TDF), zidovudine (AZT), and ritonavir-boosted lopinavir (LPV/r), efavirenz (EFV) and dolutegravir (DTG). The most popular ART regimens used in the clinic include: LPV/r based e.g. ABC + 3TC + LPV/r and DTG based e.g. TDF + 3TC + DTG.

In accordance with the clinic guidelines, all CLHIV regardless of viral load level are placed on cotrimoxazole. Cotrimoxazole is a combination of trimethoprim and sulfamethoxazole and is recommended for the prevention of Pneumocystis pneumonia, toxoplasmosis, bacterial infections, and malaria [13].

### Study Population

The study participants were CLHIV who had been on ART for at least 6 months attending the Paediatric HIV clinic at KBTH and PML.

#### Inclusion criteria

CLHIV under 15 years and who had been on ART for at least 6 months.

#### Exclusion criteria

CLHIV under 15 years who had been on ART for at least 6 months and were symptomatic for malaria at the time of recruitment.

### Sample Size Determination

Cochran’s sample size formula for prevalence studies was used to calculate the sample size using a confidence level of 95%, an error margin of 5% and a design effect of 1.5 due to the multicenter design. The prevalence of HIV-malaria co-infection (12.3%) used in the sample size calculation was from a study by [6]. The sample size obtained was 277 participants. There were 140 participants recruited from the PML and 137 participants from the KBTH.

### Sampling Method and Data Collection Tools

Participants were sampled consecutively at each site until the total number of participants needed was achieved. Blood samples were collected from each participant to detect viral load and carriage of malaria parasites. A questionnaire was designed to collect information from the study participants. The three components of the questionnaire were: the socio-demographic characteristics of the participants, malaria preventive practices, and ART-related practices. Socio-demographic data included age, gender, residence, and caregivers’ occupation and level of education. Data on malaria preventive practices of the caregiver included the use of an insecticide-treated net, mosquito coil, and mosquito repellent. Respondents were also asked whether the child was on cotrimoxazole.

The ART-related factors including the type of drug regimen, and the duration of ART were derived from the participant medical records. There was also a section that documented the viral load and malaria parasite density taken on the day of the interview. A 14-day recall by the caregiver was used to gauge adherence. Participants were questioned about their recent 14-day adherence by asking them to recollect taking their prescribed medications. Participants were questioned about their adherence during the day, the previous three days, the previous week, and the past 14 days prior to the interview. Using the formula below, the level of drug use compliance during the previous 14 days was calculated. [14]:

$$\%14dayAdherence=\frac{\left(\#\mathrm{dosesshouldhavetaken}-\#\mathrm{misseddoses}\right)}{\#\mathrm{dosesshouldhavetaken}}*100$$

From the formula, the interpretation of adherence was as follows: good adherence equivalent to ≥ 95%, fair adherence equivalent to 85–94%, and poor adherence equivalent to < 85%.

### Data Collection Procedure

Data was collected over eight weeks. Using the eligibility criteria, potential participants and their caregivers were identified, and received written and verbal information about the study, and those who were willing to take part endorsed a consent form either by signing or thumb printing. Children above the age of nine years endorsed an assent form. All children and their caregivers received a copy of the endorsed form. The questionnaire was administered using face-to-face interviews and additional information such as current drug regimen and duration of ART was extracted from the patient’s medical records. Trained research assistants thoroughly reviewed all questionnaires to validate and ensure that they had been fully completed.

### Laboratory Procedures

At the laboratory, 2ml of blood was taken from each participant: 1ml was used to measure viral load whilst ~ 100 μl was used to make thick and thin films on a microscope slide to detect malaria parasites by microscopy. DNA was extracted from the rest of the blood to detect *Plasmodium* parasites by PCR. The thin and thick blood smears were processed for Giemsa staining and evaluated using a WHO protocol [15]. *Plasmodium* species were identified after evaluating the thin films, and parasite density was estimated using the thick films. To detect satisfactory fields, the whole smear was first screened at a low magnification (10X × 40X objective lens) [15]. *Plasmodium* species identification and parasitaemia were determined using 100X oil immersion microscopy.

### Estimation of parasite density

Parasite density was determined for each malaria positive slide. The total number of white blood cells (neutrophils, eosinophils, lymphocytes and basophils) and *Plasmodium* parasites were counted in each field on the slides using a tally counter until a total of 200 white blood cells were obtained and the corresponding parasite count was recorded respectively. *Plasmodium* parasites were counted per 200 leukocytes which was used to estimate the parasite density.


$$Parasitedensity=\frac{\mathrm{TotalnumberofPlasmodiumparasite}}{\mathrm{Totalnumberofwhitebloodcells}}x8,000\mu l$$

Two independent, experienced microscopists, who were blinded to the patients’ clinical status and to the results of the microscopy examined all coded smears for parasites.

### PCR identification of Plasmodium falciparum isolates

The nested PCR method for Plasmodium species identification based on the amplification of the small unit ribosomal RNA (18SrRNA) gene was used[16]. The initial PCR involved the use of genus-specific rPLU5 and rPLU6 primers to amplify the gDNA fragment or sequence conserved in the four human Plasmodium species. The primary amplified product was used as the template for the secondary PCR using species-specific oligonucleotide primer pairs for Plasmodium species. The primer pair for *P. falciparum* was rFAL1 and rFAL2. Genomic DNA from *Plasmodium falciparum* 3D7 strain was used as a positive and double-distilled water as a negative control in all amplification reactions [17], [18].

The gene amplifications were carried out in a 15 μL reaction volume made of 200 nM dNTP, 2 mM MgCl_2_, 200 nM of each primer, and 0.5 U of One Taq DNA polymerase (New England BioLAB, UK). Four microliters (> 20 ng) of gDNA was used as a template for the primary reaction and 1 μL of the product was used as the template for the secondary reaction. The amplification cycling conditions were initial denaturation at 94°C for 5 minutes, followed by 30 cycles of 94°C for 30 seconds, 58°C annealing temperature for 1 minute, and 72°C for 1 minute with a final extension at 72°C for 5 minutes. All conditions remained the same for the primary and secondary amplifications except the secondary reaction annealing temperature which was 55°C [17], [18].

Malaria parasite detection was performed at the Immunology Department of the Noguchi Memorial Institute for Medical Research, University of Ghana.

The GeneXpert platform was used to estimate the viral load. Plasma (1.2 ml) was transferred into the Xpert HIV-1 Viral Load cartridge using a calibrated pipette and loaded into the machine. Test results were observed and recorded after 90 minutes. The limit of detection was 40 cp/ml and the clinical cut-off was 1000 cp/ml. The viral load measurements were done at the chest clinic laboratory at KBTH which participates in an external quality assurance testing program by the South African Public Health Reference Laboratory.

### Management of patients based on laboratory results

Participants who tested positive for malaria were referred to their clinician for management. Oral Artemether Lumefantrine medications were provided to the facility for dispensing to such patients. Clinicians were also made aware of the viral load level of participants. All patients were managed according to a standard protocol.

### Statistical Analysis

Standard descriptive analysis was performed using frequencies and percentages for categorical variables, means and standard deviations for normally distributed continuous variables and medians and interquartile ranges for non-normally distributed continuous variables. Pearson’s chi-squared test was used to determine the association between the carriage of malaria parasites (PCR positivity) and associated factors (socio-demographic characteristics, malaria preventive practices and ART). Pearson’s chi-square test was also used to assess the association between carriage of malaria parasites and virological suppression (target not detected). The t test was used to test the equality of means between children with malaria PCR positivity of those with malaria PCR negativity.

Finally, the binary logistic regression model was used to assess variables independently associated with Malaria PCR presence among CLHIV. In the final logistic regression model, viral load suppression was included as a major exposure variable, while age, and sex were included due to their relevance in the prevalence of health outcomes among children. Cotrimoxazole use and study site were included in the final adjusted model because of their significance level below 0.1000 in the unadjusted binary logistic regression model. Independent variables that showed a statistically significant association with the outcome variable by the univariate analysis were subjected to multivariate logistic regression. The crude odds ratio (COR) and the adjusted odds ratio (AOR) and the corresponding 95% confidence interval (CI) and p-values were presented. In the final model, statistical significance was observed with a p-values below 0.05.

### Ethical Considerations

Ethical approval to conduct research at PML was granted from the Ghana Health Service Ethical Review Board, Accra (GHS-ERC: 047/07/22), while approval to conduct the study at KBTH was given by the Institutional Review Board of Korle Bu Teaching Hospital. (KBTH-STC/IRB/000110/2022). Informed consent to do the study was obtained from the legal guardians of the study participants.

## Results

### Socio-demographic, malaria preventive practices and ART characteristics of participants

A total of 277 participants, 140 from PML and 137 from KBTH, were enrolled in the study. More than half of the participants were males (158, 57%). The age group 10–14 years had the highest number of participants 123 (41.5%). The mean age of the participants was 9.0 ± 3.4 years. The primary caregivers of the participants were mainly biological mothers, 150 (54.2%). Approximately 90% of caregivers were employed (Table 1).

In all, 26 (9.4%) participants did not practice any malaria preventive method. Out of the 277 participants, 218 (78.7%) were on cotrimoxazole. The majority of participants (224, 80.9%) had good adherence to ART. About 48% of participants had been on ART between 12 and 59 months. The ART regimens used by participants were EFV- based 104 (37.5%), followed by DTG- based 90 (32.5%), LPV/r- based 73 (26.4%) and NVP- based 10 (3.6%).

### Proportion of CLHIV with malaria parasites

Out of the 277 participants, microscopy detected 10 (3.6%, 95% CI: 1.9–6.6%) while PCR detected 21 cases of malaria (7.6%, 95% CI: 5.0–11.4%). The results are presented based on the PCR results. PCR positivity was higher among children from the PML facility (11.4%) than among the KBTH facility (3.6%).

### Viral load levels among CLHIV

**Figure 1:**
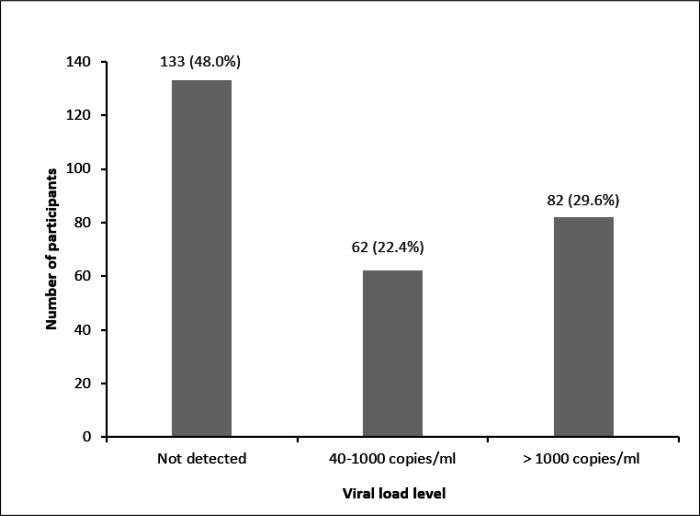
Viral load level of participants

Out of the 277 participants, 82 had virological non-suppression (VL > 1000 copies/ml). Of the participants who were suppressed, 62 (22.4%) had low-level viraemia (VL level 40–1000 copies/ml) while 133(48%) had non-detectable viral load levels. (Fig. 1).

### Malaria PCR positivity by ART regimen among participants

**Figure 2:**
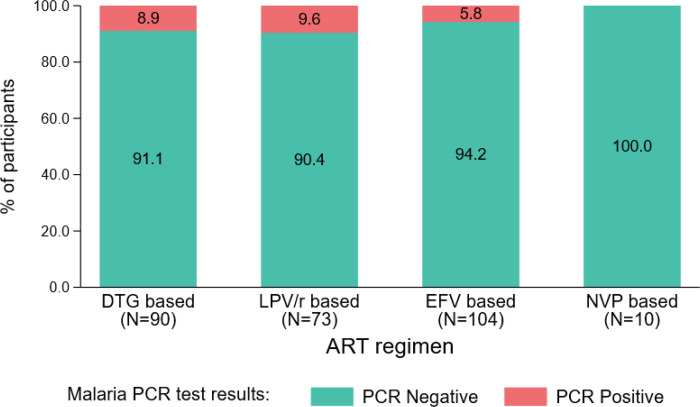
Malaria PCR positivity by ART regimen among participants

Out of the 10 patients who were on a nevirapine-based regimen, none were positive for malaria. The proportion of participants who had malaria parasites among participants on a DTG- based regimen was comparable to that among participants on a LPV/r based regimen (8.9% versus 9.6%) (Fig. 2).

There was no significant association between malaria PCR positivity and the characteristics of the participants (Table 2).

### Association between characteristics of participants and malaria PCR positivity

There was no significant association between malaria PCR positivity and the characteristics of the participants (Table 2).

### Association between characteristics of participants and virological non-suppression

The age of the child, level of adherence and ART regimen were associated with virological non-suppression. More than half of the children younger than 5 years were virologically non-suppressed (56.4%) compared to a 28.7% non-suppression among children aged 5–9 years and 22.0% non-suppression among children aged 10–14 years. Virological non-suppression was highest among children with poor medication adherence with a 60.0% prevalence, compared to a 25.9% non-suppression rate among children with good adherence and a 36.4% non-suppression rate among children with fair adherence level. Additionally, virological non-suppression rates were highest among children who had been on ART for less than 12 months (57.1%) compared to 35.1% among children with 12–59 months on ART, 20.0% among children with 60–119 months on ART and 23.5% among children with 120 or more months on ART. Virological non-suppression was highest among children on LPV/r -based regimen with 45.2% prevalence, followed by children on EFV based regimen (26.0%) then children on the DTG- based regimen (23.3%) and was lowest among children on the NVP- based regimen (10.0%) (Table 3).

### Factors associated with malaria PCR positivity by logistic regression.

The multivariate model considered the effect of sex, age of the child, use of cotrimoxazole, study site and viral load level. None of the factors were significantly associated with *P. falciparum* PCR positivity (Table 4).

### Factors associated with virological non-suppression by binary logistic regression

The multivariate model of factors associated with viral load non-suppression considered study site, age of the child, adherence to medication, ART duration and ART regimen. The odds of having non-suppression were significantly lower among the older age groups relative to those aged < 5 years with 57% lower odds among those aged 5–9 years (AOR = 0.43 [CI = 0.19–0.97], p = 0.042) and 63% lower among those aged 10–14 years (AOR = 0.37 [CI = 0.14–0.98], p = 0.046). Relative to those with good adherence to medication, the odds of viral load non-suppression were approximately five times higher among those with poor adherence (AOR = 4.89 [CI = 2.00–11.98], p = 0.001) (Table 5).

## Discussion

This study determined the proportion of asymptomatic CLHIV with malaria parasites and associated factors in paediatric HIV clinics in two hospitals in Accra, Ghana. The proportion of CLHIV with malaria was low for cases detected through microscopy and PCR. There were no factors associated with the carriage of malaria parasites. Over a third of the participants were virologically non suppressed. The odds of viral load non-suppression were approximately five times higher among those with poor adherence compared to those with good adherence.

CLHIV are at a higher risk of developing malaria parasitaemia than HIV-negative children. The higher risk of malaria parasitaemia among CLHIV could be due to immune deficiencies and dysfunction attributable to the HIV infection. Data from repeated cross-sectional surveys of school children in two ecological zones in Ghana indicates that 24% of asymptomatic HIV-negative children in Cape Coast, Ghana, had malaria parasites [19]. In this study, however, the proportion of children with malaria parasites among the CLHIV was comparatively low (3.6%). This could be because the majority of the participants were using some form of malaria preventive method. Furthermore, the majority of the participants were from urban Accra, where the transmission of malaria is relatively low. The low rate of malaria parasitaemia observed in this study (3.6%) is significantly lower than that observed in asymptomatic CLHIV patients in Douala City, Cameroon (8.8%) [12] and Benin City, Nigeria (34.1%) [13].

Trends in malaria prevalence amongst children have generally been decreasing over the past years according to the 2019 Ghana Malaria Indicator Survey [20].The percentage of children under age 5 testing positive for malaria according to microscopy decreased consistently over time, from 27% in 2014 to 21% in 2016 and 14% in 2019. The percentage of 3.6% observed in this study looks small but requires attention since malaria infection is said to claim the live of one child under 5 years of age in every 2 mins in the country [21]. Malaria prevalence in Ghana is generally on a downwards trend due to malaria interventions by the National Malaria Elimination Program (NMEP) of Ghana such as the use of Long-lasting Insecticide Treated Nets (LLNS), Indoor Residual Spraying (IRS) and case management with Artemisinin Combination Therapy drugs. These malaria interventions have resulted in reductions in the incidence and prevalence of malaria across Ghana.

There were no factors associated with the carriage of malaria parasites in this study. Although not statistically significant, asymptomatic malaria parasitaemia was almost three times more in the participants who were not on cotrimoxazole. This finding is consistent with previous studies that have established dual benefits of cotrimoxazole in reducing morbidity and mortality arising from HIV and malaria [22], [23].

In this study, older children (10–14 years age group) had higher rates of asymptomatic parasitaemia than the young children (< 5 years of age). Even though this finding was not statistically significant, young children who lack protective immunity are at highest risk of clinical disease and are more likely to be diagnosed and treated for malaria [24]. However, older children, who have acquired relatively more immunity, are more likely to harbour asymptomatic infections, which often go untreated [25].

Some ART drugs have shown antimalarial activity, including protease inhibitors such as lopinavir, ritonavir, and nonnucleoside reverse transcriptase inhibitors (NNRTIs) such as efavirenz [26]. There was however no association between the type of ART regimen and malaria parasitaemia in this study. Similarly, another multicenter study conducted in Kenya, Malawi and Uganda found that there was no difference between lopinavir/ ritonavir and NNRTI based ART on the clearance of *Plasmodium falciparum* clinical parasitaemia [27].

The ultimate goal of combination ART for People Living with HIV (PLHIV)is to achieve long-term virological suppression. There was a high rate of virological non-suppression (29.6%). This translates to a suppression rate of 70.4% which is far from the 95% target for virological suppression according to the UNAIDS framework for HIV program target milestones to be achieved by all countries in 2030. This highlights the need to closely follow and properly manage children on ART to suppress the viral load more in our setting. The rate of virological non-suppression obtained in this study is in agreement with other studies performed in Uganda (28%), Zimbabwe (30%) and Ghana (38.4%) [28]–[30]. Other studies have however reported lower rates: Ethiopia (12%) and South Africa (15%) [31], [32].

The odds of viral load non-suppression were approximately five times higher among those with poor adherence. This finding is similar to previous studies that have shown that virological non-suppression is significantly associated with poor adherence [28], [33], [34]. Poor adherence results in -therapeutic plasma ART drug concentrations, leading to resistance of one or more drugs in a given regimen, and with a possibility of cross-resistance to other drugs in the same class. optimal adherence may include missed or late doses, treatment interruptions, and discontinuations as well as therapeutic or partial dosing.

CLHIV require almost perfect levels of adherence to achieve long-lasting non-detectable viral load levels with optimal adherence to ART being the most common cause of virologic failure. Achieving the World Health Organization (WHO) goal of stopping the acquired immune deficiency syndrome (AIDS) epidemic as a public health threat by 2030 will depend on the success of current HIV treatment programs to keep viral load levels undetectable. The success will depend not only on good adherence to antiretroviral therapy but also on access to HIV treatment and retention in care [35]. There is a need to enhance adherence counselling to ensure that CLHIV in this study are adherent to their ART.

### Limitations of the study

The study design was unable to resolve whether past clinical malaria left persistent parasites or whether these individuals were parasitaemic because they were generally at a higher risk.

In conclusion, the study showed that the proportion of CLHIV with asymptomatic malaria parasitaemia was low. Approximately one-third of the participants had virological non-suppression. The odds of viral load non-suppression were approximately five times higher among those with poor adherence. The interaction between malaria parasitemia and viral replication may not be the main culprit for virological non suppression.

It is recommended that malaria preventive strategies be emphasized during counselling at routine clinics to further decrease the prevalence of malaria. Adherence counselling should be strengthened in these children to ensure that their viral load levels remain suppressed.

## Figures and Tables

**Table 1:** Socio-demographic, malaria preventive practices and ART characteristics of participants

	Total
	N = 277
Characteristics	n (%)
**Overall**	277 (100)
**Study site**	
KBTH	137 (49.5)
PML	140 (50.5)
**Sex**	
Male	158 (57)
Female	119 (43)
**Age of child**	
<5 years	39 (14.1)
5–9 years	115 (41.5)
10–14 years	123 (44.4)
**Relation of respondent to child**	
Mother	150 (54.2)
Father	43 (15.5)
Other relatives	84 (30.3)
**Employment status of caregiver**	
Unemployed	27 (9.7)
Employed	250 (90.3)
**Using any malaria preventive method**	
Yes	217 (78.3)
No	60 (21.7)
Cotrimoxazole	
Yes	251 (78.3)
No	26 (21.7)
**Adherence**	
Good	224 (80.9)
Fair	33 (11.9)
Poor	20 (7.2)
**Duration on ART**	
<12 months	14 (5.1)
12–59 months	134 (48.4)
60–119 months	95 (34.3)
120 +months	34 (12.3)
**ART regimen code**	
DTG based	90 (32.5)
LPV/r based	73 (26.4)
EFV based	104 (37.5)
NVP based	10 (3.6)

**Table 2:** Association between characteristics of participants and *P. falciparum* PCR positivity

	*P.falciparum* PCR test		
	PCR Negative	PCR Positive	P value
Characteristics	n (%)	n (%)	
**Overall**	**256 (92.4)**	**21 (7.6)**	
**Study site**			**0.014**
KBTH	132 (96.4)	5 (3.6)	
PML	124 (88.6)	16 (11.4)	
**Sex**			0.350
Male	144 (91.1)	14 (8.9)	
Female	112 (94.1)	7(5.9)	
**Age of children, Mean ± SD**	9.0 ± 3.3	8.8 ± 3.9	0.780 ^T^
**Age group of children**			0.790
<5 years	35 (89.7)	4 (10.3)	
5–9 years	107 (93.0)	8 (7.0)	
10–14 years	114 (92.7)	9 (7.3)	
Mother	136 (90.7)	14 (9.3)	
Father	39 (90.7)	4 (9.3)	
Other relatives	81 (96.4)	3 (3.6)	
**Caregiver’s highest education**			0.830
None	17 (89.5)	2(10.5)	
Primary	41 (93.2)	3 (6.8)	
JHS/JSS/Middle school	142 (93.4)	10(6.6)	
SHS or Higher	56 (90.3)	6 (9.7)	
**Employment status**			0.970
Unemployed	25 (92.6)	2 (7.4)	
Employed	231 (92.4)	19 (7.6)	
**Cotrimoxazole**			0.057
Yes	204 (94.0)	13 (6.0)	
No	52 (86.7)	8 (13.3)	
**Using any malaria preventive method**			0.12
No	27 (100.0)	0 (0.0)	
Yes	229 (91.6)	21 (8.4)	
**Adherence**			0.42
Good	208 (92.9)	16 (7.1)	
Fair	31 (93.9)	2 (6.1)	
Poor	17 (85.0)	3 (15.0)	
**Duration on ART**			0.97
<12 months	13 (92.9)	1 (7.1)	
12–59 months	124 (92.5)	10 (7.5)	
60–119 months	87 (91.6)	8 (8.4)	
120 + months	32 (94.1)	2 (5.9)	
**ART regimen code**			0.58
DTG based	82 (91.1)	8 (8.9)	
LPV/r based	66 (90.4)	7 (9.6)	
EFV based	98 (94.2)	6 (5.8)	
NVP based	10 (100.0)	0 (0.0)	

**Table 3:** Association between participant characteristics and virological non-suppression

	Viral load level		
	Suppression	Non-suppression	P value
Characteristics	n (%)	n (%)	
**Overall**	195 (70.4)	82 (29.6)	
**Study site**			**0.005**
KBTH	107 (78.1)	30 (21.9)	
PML	88 (62.9)	52 (37.1)	
**Sex**			0.46
Male	114 (72.2)	44 (27.8)	
Female	81 (68.1)	38 (31.9)	
**Age of children, Mean ± SD**	9.3 ± 3.2	8.1 ± 3.7	**0.004**
**Age group of children**			**<0.001**
<5 years	17 (43.6)	22 (56.4)	
5—9 years	82 (71.3)	33 (28.7)	
10–14 years	96 (78.0)	27 (22.0)	
**Relation of respondent to child**			0.91
Mother	104 (69.3)	46 (30.7)	
Father	31 (72.1)	12 (27.9)	
Other relatives	60 (71.4)	24 (28.6)	
**Caregiver’s highest education**			0.44
None	13 (68.4)	6 (31.6)	
Primary	28 (63.6)	16 (36.4)	
JHS/JSS/Middle school	113 (74.3)	39 (25.7)	
SHS or Higher	41 (66.1)	21 (33.9)	
**Employment status**			0.66
Unemployed	20 (74.1)	7 (25.9)	
Employed	175 (70.0)	75 (30.0)	
**Cotrimoxazole**			0.81
Yes	152 (70.0)	65 (30.0)	
No	43 (71.7)	17 (28.3)	
**Using any malaria preventive method**			0.38
No	21 (77.8)	6 (22.2)	
Yes	174 (69.6)	76 (30.4)	
**Adherence**			**0.004**
Good	166 (74.1)	58 (25.9)	
Fair	21 (63.6)	12 (36.4)	
Poor	8 (40.0)	12 (60.0)	
**Duration on ART**			**0.008**
<12 months	6 (42.9)	8 (57.1)	
12–59 months	87 (64.9)	47 (35.1)	
60–119 months	76 (80.0)	19 (20.0)	
120 + months	26 (76.5)	8 (23.5)	
**ART regimen code**			**0.005**
DTG based	69 (76.7)	21 (23.3)	
LPV/r based	40 (54.8)	33 (45.2)	
EFV based	77 (74.0)	27 (26.0)	
NVP based	9 (90.0)	1 (10.0)	

**Table 4:** Binary logistic regression model of factors associated with malaria PCR positivity among CLHIV

PCR positivity	Unadjusted model		Adjusted model	
Characteristics	COR [95% CI]	P value	AOR [95% CI]	P value
**Sex**				
Male	1.00 [reference]		1.00 [reference]	
Female	0.64 [0.25, 1.65]	0.358	0.56 [0.20, 1.55]	0.263
**Age of child**				
< 5 years	1.45 [0.42, 5.00]	0.559	0.88 [0.22, 3.59]	0.864
5–9 years	0.95 [0.35, 2.55]	0.914	0.97 [0.34, 2.75]	0.951
10–14 years	1.00 [reference]		1.00 [reference]	
**Cotrimoxazole**				
Yes	1.00 [reference]		1.00 [reference]	
No	2.41 [0.95, 6.14]	0.064	2.30 [0.88, 6.05]	0.091
**Study site**				
KBTH	1.00 [reference]		1.00 [reference]	
PML	3.41 [1.21, 9.59]	**0.020**	2.85 [0.92, 8.86]	0.070
**Suppression (Copies/ml)**				
Viral load suppression (≤ 1,000)	1.00 [reference]		1.00 [reference]	
Viral load non-suppression (> 1000)	1.88 [0.76, 4.66]	0.173	1.72 [0.63, 4.72]	0.289

**Table 5:** Binary logistic regression model of factors associated with viral load non-suppression among CLHIV

	Unadjusted model	Adjusted model		
Characteristics	COR [95% CI]	P value	AOR [95% CI]	P value
**Study site**				
KBTH	1.00 [reference]		1.00 [reference]	
PML	2.11 [1.24, 3.59]	0.006	1.47 [0.81,2.69]	0.207
**Age of child category**				
<5 years	1.00 [reference]		1.00 [reference]	
5–9 years	0.31 [0.15, 0.66]	0.002	0.43 [0.19, 0.97]	0.042
10–14 years	0.22 [0.10, 0.47]	<0.001	0.37 [0.14, 0.98]	0.046
**Adherence**				
Good	1.00 [reference]		1.00 [reference]	
Fair	1.64 [0.76, 3.54]	0.211	1.53 [0.63, 3.69]	0.347
Poor	4.29 [1.67, 11.04]	0.003	4.89 [2.00, 11.98]	0.001
**ART duration**				
<12 months	1.00 [reference]		1.00 [reference]	
12–59 months	0.41 [0.13, 1.24]	0.113	0.55 [0.20, 1.49]	0.236
60–119 months	0.19 [0.06, 0.61]	0.005	0.36 [0.12, 1.06]	0.064
120 + months	0.23 [0.06, 0.87]	0.030	0.51 [0.13, 1.92]	0.316
**ART regimen**				
DTG based	2.74 [0.33, 22.98]	0.353	3.12 [0.28, 34.80]	0.355
LPV/r based	7.43 [0.89, 61.90]	0.064	5.10 [0.45, 58.09]	0.189
EFV based	3.16 [0.38, 26.18]	0.287	2.57 [0.24, 27.88]	0.437
NVP based	1.00 [reference]		1.00 [reference]	

COR: crude odds ratio. AOR: adjusted odds ratio. CI: confidence interval

## Data Availability

The data sets used and analysed during this study are available with the corresponding author on request.

## Abbreviations

ABC: Abacavir
AIDS: Acquired Immunodeficiency Syndrome
AOR: Adjusted Odds Ratio
ART: Antiretroviral Therapy
CLHIV: Children Living with HIV
DTG: Dolutegravir
EFV: Efavirenz
HIV: Human Immunodeficiency Virus
KBTH: Korle Bu Teaching Hospital
LPV/r: ritonavir boosted lopinavir
NACP: National AIDS Control Programme
NVP: Nevirapine
PCR: Polymerase Chain Reaction
PI: Protease Inhibitor
SPSS: Statistical Package for Social Sciences
TDF: Tenofovir
COR: Crude Odds Ratio
WHO: World Health Organization
ZDV: Zidovudine
3TC: Lamivudine

## References

[1] Kwenti T. E., “Malaria and HIV coinfection in sub-Saharan Africa: prevalence, impact, and treatment strategies,” Res. Rep. Trop. Med., vol. Volume 9, 2018, doi: 10.2147/rrtm.s154501. PMC6067790 30100779

[2] Sandie S. M., Sumbele I. U. N., Tasah M. M., and Kimbi H. K., “Malaria parasite prevalence and Haematological parameters in HIV seropositive patients attending the regional hospital Limbe, Cameroon: a hospital-based cross-sectional study,” BMC Infect. Dis., vol. 19, no. 1, 2019, doi: 10.1186/S12879-019-4629-4. PMC6873725 31752719

[3] Flateau C., Le Loup G., and Pialoux G., “Consequences of HIV infection on malaria and therapeutic implications: a systematic review,” Lancet Infect. Dis., vol. 11, no. 7, pp. 541–556, Jul. 2011, doi: 10.1016/S1473-3099(11)70031-7. 21700241

[4] Alemu A., Shiferaw Y., Addis Z., Mathewos B., and Birhan W., “Effect of malaria on HIV/AIDS transmission and progression,” Parasites and Vectors, vol. 6, no. 1, pp. 1–8, Jan. 2013, doi: 10.1186/1756-3305-6-18/COMMENTS. 23327493 PMC3564906

[5] Alemu A., Shiferaw Y., Addis Z., Mathewos B., and Birhan W., “Effect of malaria on HIV/AIDS transmission and progression,” Parasit. Vectors, vol. 6, no. 1, p. 18, Jan. 2013, doi: 10.1186/1756-3305-6-18. 23327493 PMC3564906

[6] Ikilezi G. , “Short report: Prevalence of asymptomatic parasitemia and gametocytemia among HIV-infected Ugandan children randomized to receive different antiretroviral therapies,” Am. J. Trop. Med. Hyg., vol. 88, no. 4, pp. 744–746, 2013, doi: 10.4269/ajtmh.12-0658. 23358639 PMC3617863

[7] Das N. G. , “Role of asymptomatic carriers and weather variables in persistent transmission of malaria in an endemic district of Assam, India,” Infect. Ecol. Epidemiol., vol. 5, no. 1, p. 25442, Jan. 2015, doi: 10.3402/iee.v5.25442. 25595688 PMC4297276

[8] Danquah I., Ziniel P., Eggelte T. A., Ehrhardt S., and Mockenhaupt F. P., “Influence of haemoglobins S and C on predominantly asymptomatic Plasmodium infections in northern Ghana,” Trans. R. Soc. Trop. Med. Hyg., vol. 104, no. 11, pp. 713–719, Nov. 2010, doi: 10.1016/j.trstmh.2010.08.001. 20800861

[9] Greenwood B., Marsh K., and Snow R., “Why do some African children develop severe malaria?,” Parasitol. Today, vol. 7, no. 10, pp. 277–281, 1991, doi: 10.1016/0169-4758(91)90096-7. 15463389

[10] Gemechu A. , “Virological Non-Suppression among Newly Diagnosed HIV-Positive Individuals on Dolutegravir-Based Antiretroviral Treatment in Eastern Ethiopia: Follow-Up Study,” Trop. Med. Infect. Dis. 2023, Vol. 8, Page 391, vol. 8, no. 8, p. 391, Jul. 2023, doi: 10.3390/TROPICALMED8080391. PMC10458791 37624329

[11] World Health Organization, Consolidated guidelines on HIV prevention, testing, treatment, service delivery and monitoring : recommendations for a public health approach. 2021. 34370423

[12] WHO, “From malaria control to malaria elimination: a manual for elimination scenario planning,” Who, vol. 52, no. 1, p. 67, 2014, [Online]. Available: https://apps.who.int/iris/bitstream/handle/10665/112485/9789241507028_eng.pdf?sequence=1

[13] “WHO | 7.3 Monitoring response to ART and the diagnosis of treatment failure.” http://www.who.int/hiv/pub/guidelines/arv2013/art/artmonitoring/en/index3.html (accessed Jan. 11, 2017).

[14] Suleiman I. A. and Momo A., “Adherence to antiretroviral therapy and its determinants among persons living with HIV/AIDS in Bayelsa state, Nigeria,” Pharm. Pract., vol. 14, no. 1, pp. 0–0, 2016, doi: 10.18549/PHARMPRACT.2016.01.631. PMC4800010 27011771

[15] WHO, “Giemsa staining of malaria blood films,” 2016. https://www.who.int/publications/i/item/HTM-GMP-MM-SOP-07a (accessed Dec. 02, 2023).

[16] Snounou G. , “High sensitivity of detection of human malaria parasites by the use of nested polymerase chain reaction,” Mol. Biochem. Parasitol., vol. 61, no. 2, pp. 315–320, Oct. 1993, doi: 10.1016/0166-6851(93)90077-B. 8264734

[17] Ayanful-Torgby R., Quashie N. B., Boampong J. N., Williamson K. C., and Amoah L. E., “Seasonal variations in Plasmodium falciparum parasite prevalence assessed by varying diagnostic tests in asymptomatic children in southern Ghana,” PLoS One, vol. 13, no. 6, Jun. 2018, doi: 10.1371/JOURNAL.PONE.0199172. PMC6003688 29906275

[18] Adjah J., Fiadzoe B., Ayanful-Torgby R., and Amoah L. E., “Seasonal variations in Plasmodium falciparum genetic diversity and multiplicity of infection in asymptomatic children living in southern Ghana,” BMC Infect. Dis., vol. 18, no. 1, pp. 1–10, Aug. 2018, doi: 10.1186/S12879-018-3350-Z/FIGURES/4. 30157794 PMC6114730

[19] Mensah B. A., Myers-Hansen J. L., Obeng Amoako E., Opoku M., Abuaku B. K., and Ghansah A., “Prevalence and risk factors associated with asymptomatic malaria among school children: repeated cross-sectional surveys of school children in two ecological zones in Ghana,” BMC Public Health, vol. 21, no. 1, pp. 1–9, Dec. 2021, doi: 10.1186/S12889-021-11714-8/TABLES/3. 34535112 PMC8447720

[20] Statistical Service Accra G., “Ghana Malaria Indicator Survey 2019 Final Report,” 2020, Accessed: Dec. 12, 2023. [Online]. Available: www.DHSprogram.com.

[21] District J., Domechele W., Pokoanti Wak G., and Bruno Zotor F., “Prevalence and trend of malaria with anaemia among under-five children in Jasikan District, Ghana,” biorxiv.orgW Domechele, GP Wak, FB ZotorbioRxiv, 2020•biorxiv.org, doi: 10.1101/2020.03.24.005280.

[22] Okonkwo I. R., Ibadin M. O., Omoigberale A. I., and Sadoh W. E., “Effect of HIV-1 Serostatus on the Prevalence of Asymptomatic Plasmodium falciparum Parasitemia Among Children Less Than 5 Years of Age in Benin City, Nigeria,” J. Pediatric Infect. Dis. Soc., vol. 5, no. 1, pp. 21–28, Oct. 2015, doi: 10.1093/jpids/piu093. 26908488

[23] Mermin J. , “Effect of co-trimoxazole prophylaxis, antiretroviral therapy, and insecticide-treated bednets on the frequency of malaria in HIV-1-infected adults in Uganda: a prospective cohort study,” Lancet, vol. 367, no. 9518, pp. 1256–1261, Apr. 2006, doi: 10.1016/S0140-6736(06)68541-3. 16631881

[24] Carneiro I. , “Age-patterns of malaria vary with severity, transmission intensity and seasonality in sub-Saharan Africa: A systematic review and pooled analysis,” PLoS One, vol. 5, no. 2, Feb. 2010, doi: 10.1371/JOURNAL.PONE.0008988. PMC2813874 20126547

[25] Yeka A. , “Factors associated with malaria parasitemia, anemia and serological responses in a spectrum of epidemiological settings in Uganda,” PLoS One, vol. 10, no. 3, Mar. 2015, doi: 10.1371/JOURNAL.PONE.0118901. PMC4358889 25768015

[26] Kanters S. , “Comparative efficacy and safety of first-line antiretroviral therapy for the treatment of HIV infection: a systematic review and network meta-analysis,” lancet. HIV, vol. 3, no. 11, pp. e510–e520, Nov. 2016, doi: 10.1016/S2352-3018(16)30091-1. 27658869

[27] Shaffer D. , “Brief Report: No Differences Between Lopinavir/Ritonavir and Nonnucleoside Reverse Transcriptase Inhibitor–Based Antiretroviral Therapy on Clearance of Plasmodium falciparum Subclinical Parasitemia in Adults Living With HIV Starting Treatment (A5297),” J. Acquir. Immune Defic. Syndr., vol. 89, no. 2, pp. 178–182, Feb. 2022, doi: 10.1097/QAI.0000000000002839. 34693933 PMC9425486

[28] Bulage L. , “Factors Associated with Virological Non-suppression among HIV-Positive Patients on Antiretroviral Therapy in Uganda, August 2014-July 2015,” BMC Infect. Dis., vol. 17, no. 1, p. 326, 2017, doi: 10.1186/s12879-017-2428-3. 28468608 PMC5415758

[29] Makadzange A. T. , “Clinical, Virologic, Immunologic Outcomes and Emerging HIV Drug Resistance Patterns in Children and Adolescents in Public ART Care in Zimbabwe,” PLoS One, vol. 10, no. 12, Dec. 2015, doi: 10.1371/JOURNAL.PONE.0144057. PMC4678607 26658814

[30] Afrane A. K. A. , “HIV virological non-suppression and its associated factors in children on antiretroviral therapy at a major treatment centre in Southern Ghana: a cross-sectional study,” BMC Infect. Dis., vol. 21, no. 1, Dec. 2021, doi: 10.1186/S12879-021-06459-Z. PMC8330060 34340689

[31] Chandiwana N., Sawry S., Chersich M., Kachingwe E., Makhathini B., and Fairlie L., “High loss to follow-up of children on antiretroviral treatment in a primary care HIV clinic in Johannesburg, South Africa,” Medicine (Baltimore)., vol. 97, no. 29, Jul. 2018, doi: 10.1097/MD.0000000000010901. PMC6086461 30024494

[32] Yihun B. A., Kibret G. D., and Leshargie C. T., “Incidence and predictors of treatment failure among children on first-line antiretroviral therapy in Amhara Region Referral Hospitals, northwest Ethiopia 2018: A retrospective study,” PLoS One, vol. 14, no. 5, p. e0215300, May 2019, doi: 10.1371/JOURNAL.PONE.0215300. 31042743 PMC6494040

[33] Moolasart V. , “The Effect of Detectable HIV Viral Load among HIV-Infected Children during Antiretroviral Treatment: A Cross-Sectional Study,” Children, vol. 5, no. 1, p. 6, 2018, doi: 10.3390/children5010006. 29301267 PMC5789288

[34] Shumetie A., Moges N. A., Teshome M., and Gedif G., “Determinants of Virological Failure Among HIV-Infected Children on First-Line Antiretroviral Therapy in West Gojjam Zone, Amhara Region, Ethiopia,” HIV. AIDS. (Auckl)., vol. 13, p. 1035, 2021, doi: 10.2147/HIV.S334067. PMC8684387 34934365

[35] Fonsah J. Y. , “Adherence to Antiretroviral Therapy (ART) in Yaoundé-Cameroon: Association with Opportunistic Infections, Depression, ART Regimen and Side Effects,” PLoS One, vol. 12, no. 1, Jan. 2017, doi: 10.1371/JOURNAL.PONE.0170893. PMC5283684 28141867

